# FOXR2-Mediated CNS Neuroblastoma in a Pediatric Patient Treated With Multimodal Therapy Including Proton Craniospinal Irradiation: A Case Report

**DOI:** 10.7759/cureus.110887

**Published:** 2026-06-15

**Authors:** Waseem Syed, Chetan Shah, Leticia Schaefer

**Affiliations:** 1 College of Medicine, Lake Erie College of Osteopathic Medicine, Bradenton, USA; 2 Pediatric Radiology, Nemours Children's Health System, Jacksonville, USA; 3 Radiology, Nemours Children's Health System, Jacksonville, USA

**Keywords:** foxr2, mri, neuroblastoma, proton therapy, radiology

## Abstract

FOXR2-activated central nervous system (CNS) neuroblastoma is a rare embryonal tumor characterized by an aggressive nature, frequent leptomeningeal dissemination, and poor prognosis without intensive multimodal therapy. We report a four-year-old girl with multifocal FOXR2-altered CNS neuroblastoma treated with subtotal resection, multi-agent chemotherapy per ACNS0334, autologous stem cell transplant, and proton craniospinal irradiation (CSI) with focal boost. This case highlights modern management strategies and the rationale for proton therapy in reducing long-term treatment toxicity in very young children.

## Introduction

FOXR2-activated central nervous system (CNS) neuroblastoma is a recently defined molecular subtype within embryonal tumors of the CNS, formally recognized following advances in DNA methylation profiling and transcriptomic clustering that reclassified previously heterogeneous CNS neuroectodermal tumors (CNS-PNETs) into distinct subgroups [1,2]. FOXR2 activation drives oncogenic transcriptional programs involved in cell cycle progression and tumor proliferation, conferring an aggressive clinical phenotype.

These tumors predominantly arise in early childhood and are typically large supratentorial masses with ventricular involvement at presentation. Multifocal intracranial disease and leptomeningeal dissemination are common, indicating early cerebrospinal fluid (CSF) spread even when spinal imaging appears negative at diagnosis [2,3]. Radiographically, lesions are often heterogeneous with solid and cystic components, avid enhancement, and mass effect leading to hydrocephalus.

Histologically, FOXR2-driven CNS neuroblastomas are composed of small round blue cells with neuronal differentiation markers, including synaptophysin and chromogranin positivity, but may include glial or epithelial markers, such as glial fibrillary acidic protein (GFAP) and epithelial membrane antigen (EMA) [4]. This immunophenotype supports their classification within embryonal CNS tumors with neuronal lineage differentiation. The current location of these tumors can contribute to certain behavioral challenges, although these are not linked specifically to FOX2 mutations. 

Given their high risk of dissemination, management requires aggressive multimodal therapy. Standard treatment includes maximal safe resection, intensive multi-agent chemotherapy, high-dose consolidation chemotherapy with autologous stem cell rescue, and craniospinal irradiation (CSI) to address occult leptomeningeal disease [5,6]. Despite these intensive approaches, long-term survival depends heavily on effective CNS-directed therapy.

Radiation therapy, while critical for disease control, carries substantial risk in very young children, including neurocognitive decline, endocrine dysfunction, hearing loss, and secondary malignancies [7]. Proton therapy has emerged as a preferred modality in pediatric CNS tumors due to its dosimetric advantage in reducing integral dose to developing normal tissues [8].

This case illustrates the application of modern multimodal therapy, including proton CSI, in a young child with FOXR2-mediated CNS neuroblastoma and multifocal CNS involvement.

## Case presentation

A four-year-old girl with autism spectrum disorder and global developmental delay presented after the incidental detection of bilateral papilledema on routine ophthalmologic examination.

MRI of the brain and spine performed in late 2025 demonstrated a large left frontal intraventricular mass measuring 7.5 × 9.1 × 6.8 cm. The lesion extended into the bilateral frontal horns and third ventricle and was associated with obstructive hydrocephalus. In addition, a separate enhancing left thalamic lesion was identified, consistent with metastatic disease. Overall, imaging findings were consistent with a disseminated embryonal CNS tumor [5]. This is presented in Figure 1.

**Figure 1:**
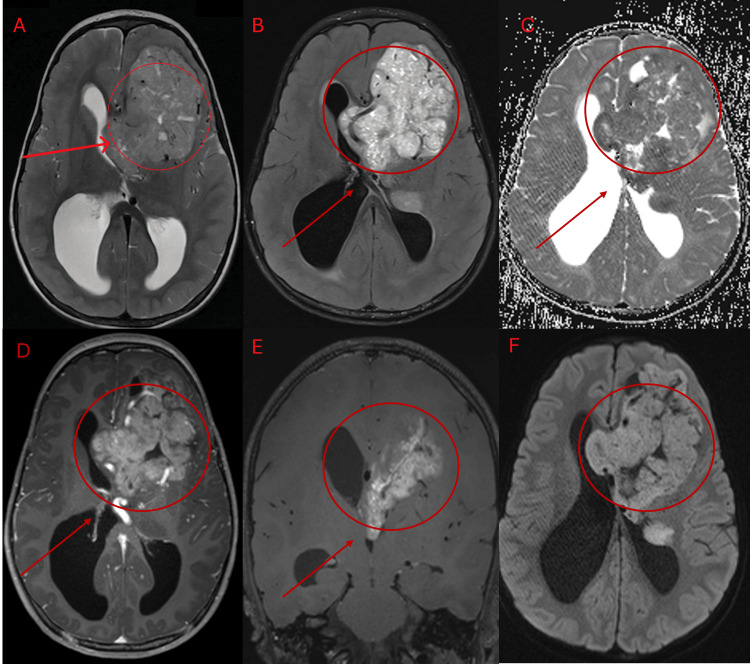
Pre-treatment multi-sequence MRI of intracranial neoplasm Multi-sequence axial and coronal magnetic resonance imaging (MRI) of the brain demonstrates a large, lobulated mass (circled) in the left cerebral hemisphere prior to treatment. A. Axial T2-weighted imaging shows a heterogeneously hyperintense mass causing significant midline shift and compression of the left lateral ventricle. B. Axial FLAIR imaging highlights the internal architecture of the lesion and associated peritumoral edema. C. Axial ADC map demonstrates areas of low signal within the mass (indicated by arrow), representing restricted diffusion. D. Axial post-contrast T1-weighted imaging reveals patchy, irregular enhancement of the solid tumor components. E. Coronal post-contrast T1-weighted imaging illustrates the vertical extent of the tumor and its displacement of midline structures. F. Axial diffusion-weighted imaging (DWI) shows corresponding hyperintensity within the mass, consistent with high cellularity.

Subtotal surgical resection revealed a small, round, blue cell tumor. Immunohistochemistry was positive for OLIG2, synaptophysin, and chromogranin, and negative for GFAP and EMA. Molecular analysis confirmed FOXR2 activation. These findings supported the diagnosis of FOXR2-activated CNS neuroblastoma [1,6]. Imaging obtained after initial tumor debulking is depicted in Figure 2.

**Figure 2:**
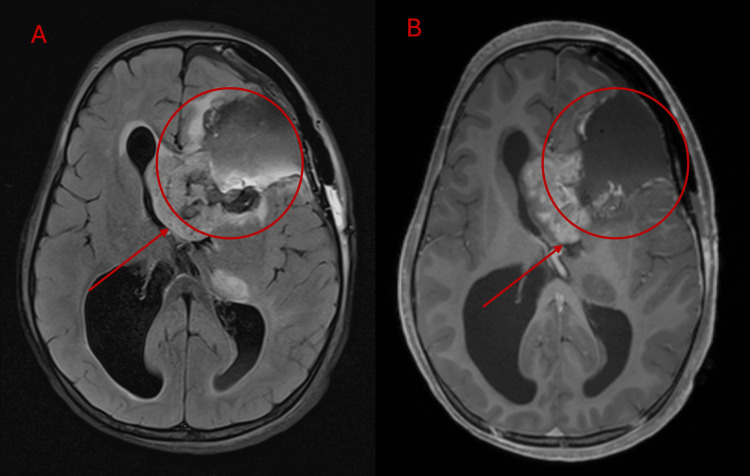
Post-operative MRI following tumor debulking Axial MRI sequences obtained after surgical intervention demonstrate the interval changes following partial resection of the left-sided mass. A. Axial FLAIR imaging shows a significant reduction in tumor volume, with a new post-surgical fluid collection/resection cavity (circled) and a decrease in the previously noted midline shift. Persistent hyperintensity at the surgical margins (arrow) likely represents residual tumor and post-operative changes. B. Axial post-contrast T1-weighted imaging reveals the extent of the resection cavity (circled) and highlights areas of residual enhancing tumor along the medial and posterior margins of the surgical bed (arrow). These images serve as the baseline for subsequent chemotherapy and radiation therapy.

The patient underwent preoperative embolization of the left anterior cerebral artery (ACA) and lenticulostriate vessels, followed by a left frontotemporal craniotomy and subtotal resection of approximately 50% of the tumor. Subtotal resection is commonly encountered in this disease due to deep ventricular involvement and vascular encasement.

The patient received induction chemotherapy per the ACNS0334 protocol, followed by consolidation high-dose chemotherapy and autologous stem cell transplant. The treatment course was complicated by febrile neutropenia, mucositis, weight loss, and dependence on nasogastric tube feeding. This intensive chemotherapy approach is consistent with current management strategies for high-risk embryonal CNS tumors [8].

Post-therapy MRI six months later demonstrated a decrease in the size of the primary tumor cavity and a reduction in subependymal and thalamic lesions. Cerebrospinal fluid cytology was negative for malignant cells. These findings were consistent with a partial response; however, the patient remained at risk for residual microscopic disease [9]. These findings are depicted in Figure 3.

**Figure 3:**
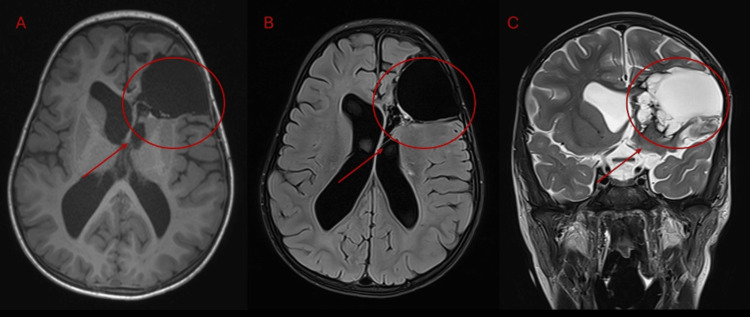
Follow-up MRI showing complete treatment response Multi-sequence follow-up MRI obtained after the completion of treatment (surgical resection and chemotherapy/radiation). A. Axial T1-weighted image showing no residual enhancing disease at the original site (circled). B. Axial FLAIR image and C. Coronal T2-weighted image showing a well-defined, cerebrospinal fluid-filled resection cavity (circled) with no surrounding mass or edema. Stable ventricular dilation is also noted, and the original midline shift (arrow) is resolving.

Given the presence of residual disease, multifocal CNS involvement, and a high risk of cerebrospinal fluid dissemination, the patient was planned for CSI with proton therapy [10].

The radiation plan consisted of CSI to a dose of 23.4 GyRBE delivered in 13 fractions, followed by a focal boost to the tumor bed and residual disease to an additional 30.6 GyRBE delivered in 17 fractions, for a total dose of 54 GyRBE in 30 fractions.

Proton therapy was selected to reduce radiation exposure to critical developing structures, including the temporal lobes, hypothalamic-pituitary axis, cochlea, and spinal and abdominal organs. This reduction in integral dose is particularly important in very young children, in whom neurocognitive and endocrine systems are highly sensitive to radiation injury [4,11].

## Discussion

FOXR2-activated CNS neuroblastoma represents a recently defined embryonal tumor entity with aggressive clinical outcome and early leptomeningeal dissemination. Molecular classification has significantly refined diagnostic accuracy and treatment stratification in pediatric neuro-oncology [1,2].

A key feature of this tumor is its high propensity for CSF dissemination. Even in cases where spinal imaging is initially negative, occult microscopic disease is common, necessitating CSI as a core component of therapy [5,10]. This biological behavior aligns FOXR2 tumors with other high-risk embryonal CNS tumors such as medulloblastoma, although FOXR2 tumors often present with larger primary tumor burden and more extensive ventricular involvement at diagnosis [3].

Surgical management is primarily aimed at maximal safe cytoreduction. However, complete resection is rarely feasible due to deep ventricular extension, vascular involvement, and proximity to eloquent brain structures. In this case, subtotal resection achieved partial debulking, but residual disease remained, consistent with previously reported surgical outcomes in embryonal CNS tumors [7].

High-dose chemotherapy with autologous stem cell rescue has become an important strategy in high-risk pediatric CNS tumors, allowing dose intensification beyond conventional toxicity thresholds. This approach has demonstrated improved disease control in select embryonal tumor populations [8].

Despite systemic therapy, CSI remains essential due to the high risk of leptomeningeal relapse. CSI significantly improves disease control in embryonal CNS tumors by addressing both macroscopic and microscopic CSF disease [10]. However, its use in very young children introduces significant concerns regarding late toxicity.

Neurocognitive impairment is one of the most clinically significant late effects of pediatric brain irradiation. Declines in IQ, executive function, and processing speed are well documented, particularly in children treated before age 7 [12]. Endocrinopathies resulting from hypothalamic-pituitary irradiation and cochlear dose-related hearing loss further contribute to long-term morbidity [11,12].

Proton therapy significantly reduces radiation exposure to normal tissues due to its Bragg peak dose distribution. In CSI, this results in lower integral brain dose and reduced exposure to thoracic, abdominal, and pelvic organs compared with photon therapy [11]. These dosimetric advantages translate into clinically meaningful reductions in predicted late toxicity.

In this patient, proton therapy was selected to minimize dose to the developing brain, cochlea, and endocrine structures, which is particularly important given her young age and baseline neurodevelopmental delay. Additionally, proton therapy may reduce the risk of radiation-induced secondary malignancies, which is a critical consideration in long-term pediatric cancer survivorship [11,12].

This case underscores the importance of integrating molecular tumor classification, aggressive multimodal therapy, and advanced radiation techniques to optimize both survival and long-term functional outcomes in rare pediatric CNS tumors.

## Conclusions

FOXR2-activated CNS neuroblastoma is a rare, highly aggressive embryonal brain tumor with a strong tendency for early dissemination. This case supports the use of a comprehensive multimodal treatment approach, including maximal safe resection, intensive chemotherapy with stem cell rescue, and CSI.

Importantly, it highlights the role of proton therapy in modern pediatric neuro-oncology. While survival remains the primary goal, minimizing long-term treatment-related morbidity is equally critical in very young children. Proton CSI provides a significant reduction in radiation dose to developing neural, endocrine, and sensory structures, thereby expected to improve the long-term therapeutic ratio.

As molecular diagnostics continue to refine tumor classification, treatment paradigms must evolve in parallel to integrate precision oncology with precision radiation delivery, optimizing both cure and survivorship outcomes.
